# Renoprotection of Microcystin-RR in Unilateral Ureteral Obstruction-Induced Renal Fibrosis: Targeting the PKM2-HIF-1α Pathway

**DOI:** 10.3389/fphar.2022.830312

**Published:** 2022-06-09

**Authors:** Yan Ren, Jie Wang, Wenwen Guo, Jingwen Chen, Xin Wu, Shubo Gu, Lizhi Xu, Zhiwei Wu, Yaping Wang

**Affiliations:** ^1^ Department of Medical Genetics, Nanjing University School of Medicine, Nanjing, China; ^2^ Jiangsu Key Laboratory of Molecular Medicine, Nanjing University School of Medicine, Nanjing, China; ^3^ Jiangsu Key Laboratory of Molecular and Translational Cancer Research, Jiangsu Cancer Hospital, Jiangsu Institute of Cancer Research, The Affiliated Cancer Hospital of Nanjing Medical University, Nanjing, China; ^4^ Department of Pathology, The Second Affiliated Hospital of Nanjing Medical University, Nanjing, China; ^5^ Center for Public Health Research, Nanjing University School of Medicine, Nanjing, China

**Keywords:** microcystin-RR (MC-RR), renoprotection, renal fibrosis, PKM2, HIF-1α

## Abstract

Renal fibrosis is a pathological characteristic of the endpoint of chronic kidney disease (CKD), which remains a major public health problem. None of the current therapies is effective in stopping kidney fibrosis progression. In light of our novel detection of a potential antifibrosis of microcystins (MCs), we investigate the renoprotection effect of MCs with UUO-induced renal fibrosis. The treatment of MCs was initiated in model animals in advance of UUO operation. After determining that the antifibrotic effect of MCs was independent of its toxicity, our study focused on the renoprotection of microcystin-RR (MC-RR), a lower toxic congener of MCs, in UUO mice and the cell models *in vitro*. The co-immunoprecipitation assay and recombination plasmid transfection were used in the investigation of the mechanism of antifibrosis of MC-RR*.* The data show that MC-RR substantially exerts an effect on renoprotection with suppression of the expression of TGF-β1/Smad signaling molecules and a blockage in epithelial dedifferentiation and myofibroblast activation in UUO model animals. MC-RR shows a binding directly to pyruvate kinase M2 (PKM2), downregulates PKM2-HIF-1α signaling, restores the inhibited expression of MMP-7 and MMP-13, and reduces the upregulated expression of MMP-9 in UUO renal tissues. The current study demonstrates a novel effect of MC-RR on renoprotection in kidney damage, which could be conducted in therapeutics for chronic kidney disease.

## Introduction

Chronic kidney disease (CKD) remains a major public health problem with a high prevalence estimated at about 10% of adults with some degree of CKD in the world population (Romagnani et al., 2017). Renal interstitial expansion is the characteristic histologic predictor of renal functional decline in CKD. The initial deposition of the fibrotic matrix after injury may be beneficial for the tissue repair process, but the continuous deposition of the fibrotic matrix during chronic injury that presents in CKD will eventually progress to end-stage renal disease. None of the current therapies is effective to resolve the lesion of renal fibrosis and stop this disease progression (Webster et al., 2017; Ruiz-Ortega et al., 2020).

Despite a varied initial evolution related to etiology diversity, for example, pre-existing diabetes, hypertension, autoimmune-related infections, poisons, and genetic susceptibility, a similar pathophysiologic process underlies the occurrence of renal fibrosis, involving inflammatory, macrophage infiltration and abnormal expression of inflammatory factors, myofibroblast differentiation and proliferation, and increased extracellular matrix (ECM) deposition (Nogueira et al., 2017). A big body of evidence implicates TGF-β1/Smad as an important signal pathway and myofibroblasts as critical effector cells in mediating renal fibrosis. Moreover, macrophage polarization has been elucidated in the progression from kidney inflammatory injury to renal fibrosis (Humphreys, 2018; Tang et al., 2019).

Recently, great attention has been paid to the metabolic perturbation involved in the pathogenesis of organ fibrosis diseases (Maher, 2015). Xie et al. (2015) demonstrated that the augmentation of aerobic glycolysis, known as the Warburg effect, was an early and sustained event during the activation of myofibroblast (Xie et al., 2015). The switch of metabolism from oxidative phosphorylation to aerobic glycolysis was also observed as the primary metabolic feature of the myofibroblast in renal fibrosis. The suppression of glycolysis could significantly alleviate renal fibrosis (Ding et al., 2017; Wei et al., 2019). A growing body of evidence indicates that aerobic glycolysis exerts effects on numerous aspects of cellular function, including energy production, cell proliferation, ECM production, autophagy, and apoptosis (Dayton et al., 2016). Pyruvate kinase M2 (PKM2) is the final rate-limiting enzyme of glycolysis and is preferentially expressed during embryonic development and in proliferating cells (Israelsen et al., 2013). PKM2 with allosteric regulation exists as either a dimeric in a low glycolytic enzyme activity or a tetramer in a high activity. The cytosolic PKM2 dimers are in equilibrium with tetramers which are retained in the cytoplasm. The dimeric PKM2 can be phosphorylated tyrosine residue and then translocate into the nucleus, where it will directly interact with hypoxia inducible factor 1 subunit alpha (HIF-1α), a master regulator for the expression of genes involved in the hypoxia response and contribute to cell proliferation, myofibroblast differentiation, and sustainment, which can result in the development of fibrosis. This mechanism highlights a potential new approach for therapeutics of fibrotic disease (Tamada et al., 2012).

Microcystins (MCs) are a common class of toxins produced by cyanobacterial blooms in various eutrophic inland waters. The known more than 100 congeners of MCs share the basic structure of monocyclic heptapeptide but with greatly variable toxicity. The difference in compositional amino acids of these congeners frequently occurs in the second and fourth amino acids. Microcystin-LR (MC-LR) in which the second and fourth are leucine (leucine, L) and arginine (arginine, R), respectively, is a more toxic congener of MCs, while MC-RR in which both are arginine (arginine, R) is less toxic (Kordasht et al., 2010; Dittmann et al., 2013; Gupta et al., 2013). We previously described that both MC-LR and MC-RR can ameliorate the pulmonary fibrosis of model animals, indicating the antifibrotic effect is independent of their toxicological mechanism (Wang et al., 2020; Wang et al., 2021). Seeing that the pathophysiologic principles underlying fibrogenesis are shared by fibrotic diseases, we hypothesize a possible antifibrotic effect of MCs on renal fibrosis (Distler et al., 2019). In view of the potential application in translational medicine, we confirmed the renoprotection of MC-LR and MC-RR and then focused on the mechanism with the less toxic MC-RR in unilateral ureteral obstruction (UUO) kidneys in the current study. We demonstrated that MC-RR substantially exerts an effect on renoprotection with the alleviation of the main characteristic phenotypes of UUO-induced renal fibrosis. Moreover, we unveiled that MC-RR had an interaction with PKM2 that was obviously up-regulated in renal tissues of UUO animals and inhibited PKM2-HIF-1α pathway–associated fibrogenesis, which was reasonably involved in the antifibrotic mechanism of MC-RR.

## Materials and Methods

### Chemicals

MC-RR (ALX-350-043) and MC-LR (ALX-350-012) were purchased from Enzo Life Sciences (Farmingdale, New York, United States). Human TGF-β1 (AF-100-21C-100) was purchased from Pepro Tech (Rocky Hill, New Jersey, United States), mouse/rat TGF-β1 (CK33) was purchased from Novoprotein (Shanghai, China), and TEPP-46 (S7302) was obtained from Selleck Chemicals (Houston, Texas, United States).

### Mouse Model

Six–eight-week-old C57BL/6 mice (20-24 g) were obtained from the Model Animal Research Center of Nanjing University (Nanjing, China). The animal experiments in the current study were approved by the Ethics Committee for Animal Research of Nanjing University School of Medicine (Nanjing, China) and conducted in accordance with the school Guide for the Care and Use of Laboratory Animals. The animals were housed in a special pathogen-free and temperature-controlled (23 ± 2°C and 50% relative humidity) with 12 h light/dark cycle, and water and chow were freely available. Three batches of animals that were obtained for constructing the UUO model by the left ureteral ligation of mice in an anesthesia state in the current study. The first batch included 40 mice that were divided into 4 groups, Sham, UUO, UUO + MC-LR (20 μg/kg), and UUO + MC-RR (20 μg/kg) groups, each with 10 mice. The intragastric administration with normal saline, MC-LR (20 μg/kg/day), or MC-RR (20 μg/kg/day) was started 28 days (4 weeks) prior to UUO operation. The second batch contained 50 mice that were intragastrically administered with MC-RR (20 μg/kg/day) in three different start times, 0 day, 14, and 28 days, respectively, prior to UUO operation. The animals were divided into 5 groups, Sham, UUO, UUO + MC-RR (0 day), UUO + MC-RR (-14 days), and UUO + MC-RR (−28 days) groups, each with 10 mice. The third batch contained 25 mice that were administered with MC-RR in three different doses, 5 μg/kg/day, 10 μg/kg/day, and 20 μg/kg/day, respectively, and was started at 28 days prior to UUO operation. The animals were divided into 5 groups, Sham, UUO, UUO + MC-RR (5 μg/kg), UUO + MC-RR (10 μg/kg), and UUO + MC-RR (20 μg/kg) groups, each with 5 mice. The administration of MC-RR lasted until day 7 after UUO, when the experimental animals were anesthetized by the intraperitoneal injection of pentobarbital sodium. The blood samples were obtained by heart punctures for the measurement of serum biochemistry. Experimental animals were killed humanely after injecting with anesthesia. The ligated kidney tissues of the model mice were collected. Part of kidney tissue of each mouse was fixed in 10% formalin overnight and the remaining tissue was kept at −80°C.

### Histology and Staining

The fixed kidney tissues were embedded in paraffin. Consecutive 5-micron sections were prepared for hematoxylin and eosin (H&E), Masson’s Trichrome, Sirius Red, and EVG staining as previously described. H&E staining is used to assess kidney tissue damage and destruction of normal kidney architecture, and Masson’s Trichrome, Sirius Red, and EVG staining are used to identify the interstitial deposition of fibrosis. H&E- and Masson-stained slides were examined under a light microscope in a double-blind manner by two histopathologists.

### Immunohistochemical Analysis

Immunohistochemistry for the protein expression of mouse kidneys was assessed using the streptavidin–peroxidase immunohistochemical method. For immunohistochemistry staining, deparaffinized sections were boiled in citrate buffer for antigen retrieval. Then, the sections were permeabilized in 1× PBS containing Triton X-100 (0.1%) for 10 min and incubated with anti-α smooth muscle actin (α-SMA, GB11044, Servicebio, Wuhan, China), antifibronectin (GB112093, Servicebio), anticollagen I (PB0981, BOSTER, Wuhan, China), anti-PKM2 antibody (4053S, CST, Boston, United States), anti-p-PKM2 (PA5-105498, Invitrogen, California, United States), anti-CD206 (ab125028, Abcam, Cambridge, United Kingdom), anti-inducible nitric oxide synthase (iNOS, ab178945, Abcam) at 4°C overnight. Immunohistochemical staining was performed using an UltraSensitive S-P kit (kit 9706, Maixin-Bio, Jinan, China). The selected regions were captured under a light microscope.

### Cell Culture

The cell lines, HK-2 (a human proximal tubular cell line derived from the normal kidneys), and NRK-49F (a rat fibroblast cell line derived from the normal kidneys) were purchased from the Cell Bank of the Typical Culture Preservation Committee, Chinese Academy of Sciences. Both cells were tested negative for *mycoplasma* contamination using a MycoBlue^TM^ *mycoplasma* detector (Vazyme, Nanjing, China). The cells were cultured in humidified air at 37°C with 5% CO_2_. HK-2 was grown in a DMEM/F12 medium (11330032, Gibco, New York, United States) and NRK-49F in DMEM medium (11965092, Gibco). All mediums were supplemented with 10% fetal bovine serum (10100147C, Gibco), 100 U/ml penicillin, and 100 μg/ml streptomycin. To induce epithelial to mesenchymal transition and fibroblast to myofibroblast transition *in vitro*, HK-2 and NRK-49F cell lines were grown in 6-well plates at a seeding density of 1×10^5^ cells/ml in the medium with TGF-β1 (5 ng/ml) for 48 h. MC-RR (0.1 μM) was synchronously added to observe the intervention effect on the induced cells.

### MTT Assay

Renal cells (HK-2 and NRK-49F) were assessed for cell viability using the MTT assay following the manufacturer’s instructions (Beyotime Biotechnology, Nanjing, China). The cells were treated with the indicated concentrations of MC-RR for 24, 48, and 72 h, respectively. The cells were incubated using MTT for 4 h at 37°C, and then the formazan lysis solution was added for 20 min. MTT absorbance was determined by spectrophotometry at 540 nm. Cell viability was calculated using the following formula: viability (%) = (OD test/OD control) ×100.

### Western Blot

Kidney tissues or cultured cells were homogenized in RIPA buffer with proteinase inhibitors and the protein extracts were boiled in SDS loading buffer. The equal amounts of samples were separated by 10% SDS-poly acrylamide gel electrophoresis (SDS-PAGE) and transferred to 0.45 μm PVDF membranes (Millipore, Germany). After blocking with 5% milk-TBST for 2 h, the PVDF membranes were incubated with the primary antibodies at 4°C overnight. The used antibodies included anti-fibronectin (ab32419, Abcam), anti-collagen I (PB0981, BOSTER), anti-α-SMA (19245S, CST), anti-E-cadherin (3195S, CST), anti-vimentin (5741S, CST), anti-snail (3879T, CST), anti-TGF-β (3709S, CST), anti-p-Smad3 (9520T, CST), anti-Smad3 (9523T, CST), p-Ser473-AKT (4060S, CST), anti-AKT (4691T, CST), anti-signal transducer and activator of transcription 6 (STAT6, 5397S, CST), anti-PKM2 (4053S, CST), anti-p-PKM2 (PA5-105498, Invitrogen), anti-HIF-1α (14179S, CST), anti-matrix metallopeptidase 7 (MMP-7, 71031S, CST), anti-matrix metallopeptidase 9 (MMP-9, 13667S, CST), anti-matrix metallopeptidase 13 (MMP-13, 69926S, CST), anti-CD206 (ab125028, Abcam), anti-iNOS (ab178945, Abcam), anti-arginase-1 (Arg1, 93668T, CST), anti-chitinase-like 3 (Ym1, ab192029, Abcam) and anti-β-actin (4970S, CST). The PVDF membranes were then washed with TBS-T (TBS with 0.1% [v/v] Tween 20) three times and incubated with the secondary antibody for 1 h at room temperature. The bands were detected using an enhanced chemiluminescence (ECL) detection system and analyzed using Quantity One software.

### Immunofluorescence Assay

The cells were fixed with 4% paraformaldehyde for 30 min at room temperature. After washing with PBS three times, the fixed cells were blocked in 3% BSA supplemented with 0.1% Triton X-100 for 1 h. Then, the cells were incubated with the primary antibody. The used antibodies included anti-PKM2 (1:300, 4053S, CST), anti-p-PKM2 (1:300, PA5-105498, Invitrogen), and anti-MC-RR (1:100, ALX-804-320, Enzo) overnight at 4°C followed by incubation with Alexa Fluor 594 or 488-conjugated IgG for 1.5 h at room temperature. The nucleus was labeled with DAPI. The images were acquired under an Olympus confocal microscope.

### Co-Immunoprecipitation (Co-IP) Assay

The cultured NRK-49F cells pretreated with TGF-β and MC-RR were rinsed twice with PBS and lysed in ice-cold immunoprecipitation buffer–containing protease inhibitor cocktail and phosphatase inhibitors on ice for 30 min, followed by centrifugation at 12000 *g* for 10 min. The supernatants were incubated with anti-PKM2 or IgG (control) overnight at 4°C and then precipitated by the protein A/G PLUS-Agarose beads for another 2 h. The beads were extensively washed and used for western blot analysis.

### Plasmid Recombination and Transfection

The recombinant PKM2 plasmid was constructed by ligating the full-length open reading frame (cDNA) of PKM2 and cloning it into an expression vector pcDNA3.1 (HanhengBio, Shanghai, China). The expression plasmids were transiently transfected into cultured NRK-49F cells with 60–80% of confluence using lipofectamine 3000 (L3000015, Thermo Fisher Scientific, Massachusetts, United States), in accordance with the manufacturer’s instructions. The transfected cells were cultured with MC-RR treatment for 48 h and harvested for the PKM2 rescue assay.

### Molecular Docking Study

The works of molecular docking were conducted using the Yinfo Cloud Platform (https://cloud.yinfotek.com/). The chemical 2D structure of MC-RR from https://pubchem.ncbi.nlm.nih.gov/was drawn using JSME (JavaScript Molecule Editor) and converted to the 3D structure with energy minimization in the MMFF94 force field. The crystal/NMR structure of the PKM2 protein (PDB code: 6JFB, resolution: 2.12 Å) was downloaded from the RCSB Protein Data Bank (http://www.rcsb.org/). The crystal ligand was separated and used to define the binding pocket. AutoDock Vina program was utilized to perform semiflexible docking with maximum 9 pose output after internal clustering.

### Microscale Thermophoresis

Recombinant human PKM2 (ab89364, Abcam) was labeled with a fluorescent dye (Pico-RED) using Monolith™ RED-NHS Protein Labeling Kits (MO-L011). The labeling procedure and the subsequent removal of free dye were performed according to the manufacturer’s instructions. The concentration of PKM2 was kept constant at 20 nM. A serial dilution of MC-RR was prepared and mixed with PKM2 by 1:1 (v/v). The highest concentration of MC-RR was 200 μM. The mixed samples were loaded into standard treated MO NT.115 capillaries (MO-K022, NanoTemper). Binding measurements were conducted using a NanoTemper Monolith NT.115 Instrument (NanoTemper Technologies GmbH) and carried out when LED power was set to medium and laser power set to 40%. The MST data of independent measurements were processed using Prism GraphPad software to calculate the binding coefficient (Kd) for interacting partners.

### ELISA

The contents of free MC-RR and MC-LR in kidney tissues were measured using the microcystin-ADDA ELISA kit (Enzo Life Sciences) which is an indirect competitive ELISA for the congener-independent detection of MCs. Following the manufacturer’s instruction, 50 mg of each tissue was lysed to release the toxins. The color intensity of the ELISA test was evaluated by absorbance at 450 nm on an M2e microtiter plate reader. The contents of MC-RR or MC-LR were calculated using the standard curve run in tested tissues.

### Serum Biochemistry

The serums were isolated from the mouse blood samples for measuring the hematological parameters. Serum parameters, including alanine aminotransferase (ALT), aspartate aminotransferase (AST), total protein (TP), total bilirubin (TB), blood urea nitrogen (BUN), and creatinine (CRE) were assayed using an Auto-dry Chemistry Analyzer (Kehua Bioengineering, Shanghai, China).

### Statistical Analysis

Statistical analysis was performed using Prism 6.00 (GraphPad Software, San Diego, CA, United States) and SPSS version 20 (SPSS, Inc., Chicago, IL, United States). The data are expressed as the means ± standard errors of the mean (SEMs). To determine the differences among multiple comparisons, the results were evaluated by the one-way analysis of variance (ANOVA) with Student–Newman–Keuls *post hoc* analysis. For the comparisons of MC-LR and MC-RR concentration in mice renal tissues, data were analyzed using an independent Student’s *t*-test. The significance level was set to *p* < 0.05.

## Results

### MC-LR and MC-RR Present a Significant Renoprotection Effect in the Mice With UUO-Induced Renal Fibrosis

**FIGURE 1:**
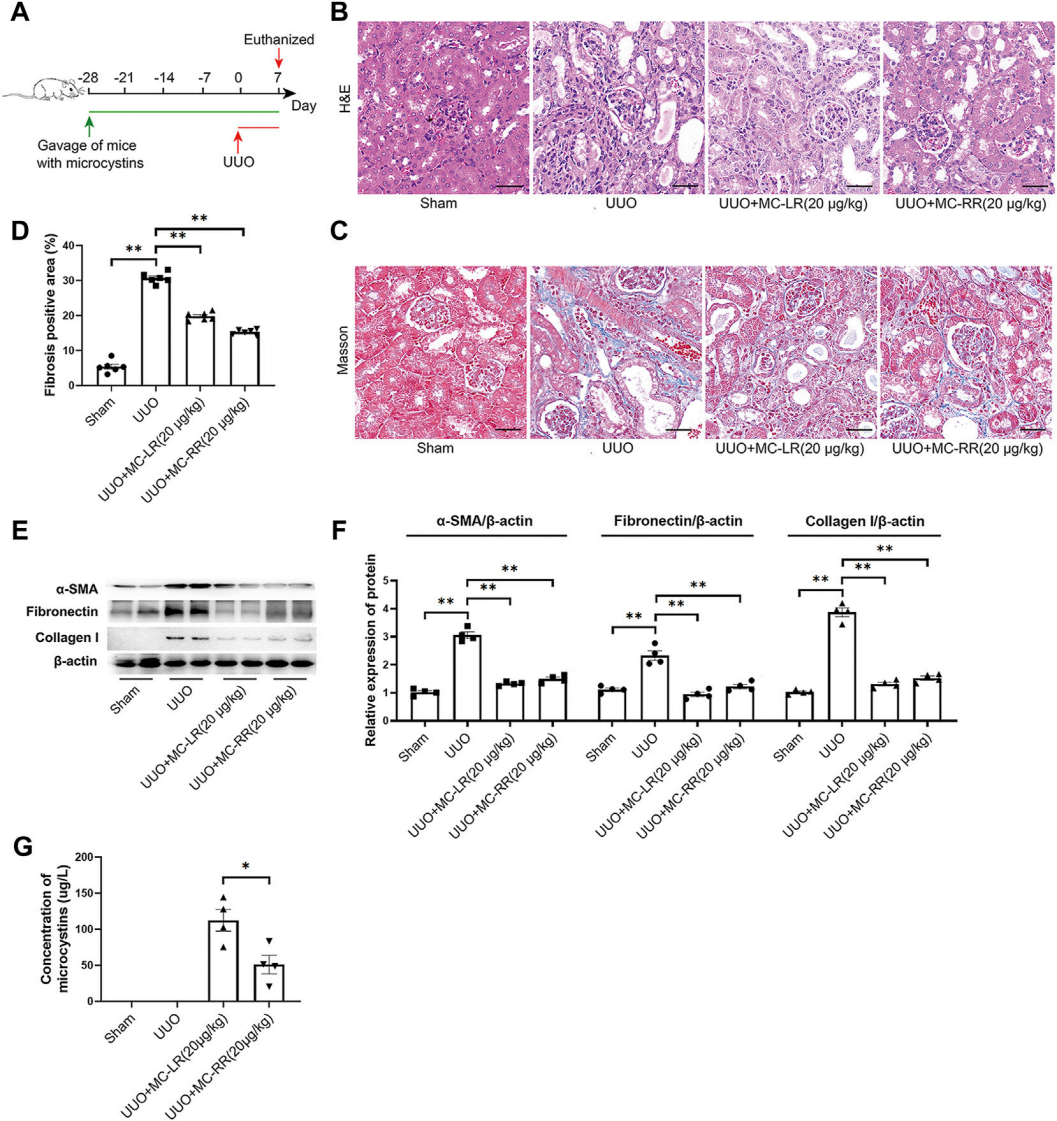
Antifibrotic effect of microcystin (MC) in renal fibrosis mice. Mice were treated with MC-LR (20 μg/kg/day) or MC-RR (20 μg/kg/day) by intragastrical administration for 4 weeks in advance, and then unilateral ureteral ligation was performed to construct a mouse model of obstructive renal fibrosis. The operated mice were administrated with MC-LR or MC-RR for another week, and then were euthanized for further analysis. **(A)** Schematic diagram of the experimental design. Forty mice were divided into four groups, Sham, unilateral ureteral obstruction (UUO), UUO + MC-LR, and UUO + MC-RR groups, each with 10 mice. No mice died during the experiment. **(B,C)** Kidney tissue sections were employed for H&E and Masson staining (scale bar: 40 μm). **(D)** Fibrosis positive area in Masson staining were assessed by pathologist blind to this study (*n* = 6). **(E,F)** The protein level of *α*-smooth muscle actin (α-SMA), fibronectin and collagen I were measured in the obstructive renal tissues of UUO mice by western blot. *p < 0.05, **p < 0.01 determined by one-way ANOVA with S–N–K *post hoc* analysis. **(G)** Residual contents of MC were detected in the kidney tissues of model mice treatment with MC-LR or MC-RR using the ELISA method. Data were analyzed using independent Student’s *t*-test, *p < 0.05.

We established the UUO model and initiated the intervention of MC-LR or MC-RR 4 weeks in advance, and continued until one week after UUO operation (Figure 1A). The changes in the body weight and kidney weight coefficient in the model mice are shown in Supplementary Figure S1. As expected, extensive structural damage, interstitial expansion, and collagen deposition were observed in the kidney tissues of UUO mice when compared with the sham group. Both MC-LR and MC-RR treatment significantly alleviated the renal lesions (Figures 1B–D). Western blot revealed a significantly increased expression of the markers of myofibroblasts, including *α*-SMA, fibronectin, and collagen I in UUO mice, while MC-LR or MC-RR significantly reduced the levels of these proteins (Figures 1E,F). This result was supported by the assay of immunohistochemical staining (Supplementary Figure S2). Interestingly, there was a lower residual content of MC-RR than that of MC-LR in the kidney tissues with the comparison between the UUO mice treated with MC-LR and MC-RR (Figure 1G).

### MC-RR Alleviates Renal Fibrosis Even in a Lower Dose

**FIGURE 2:**
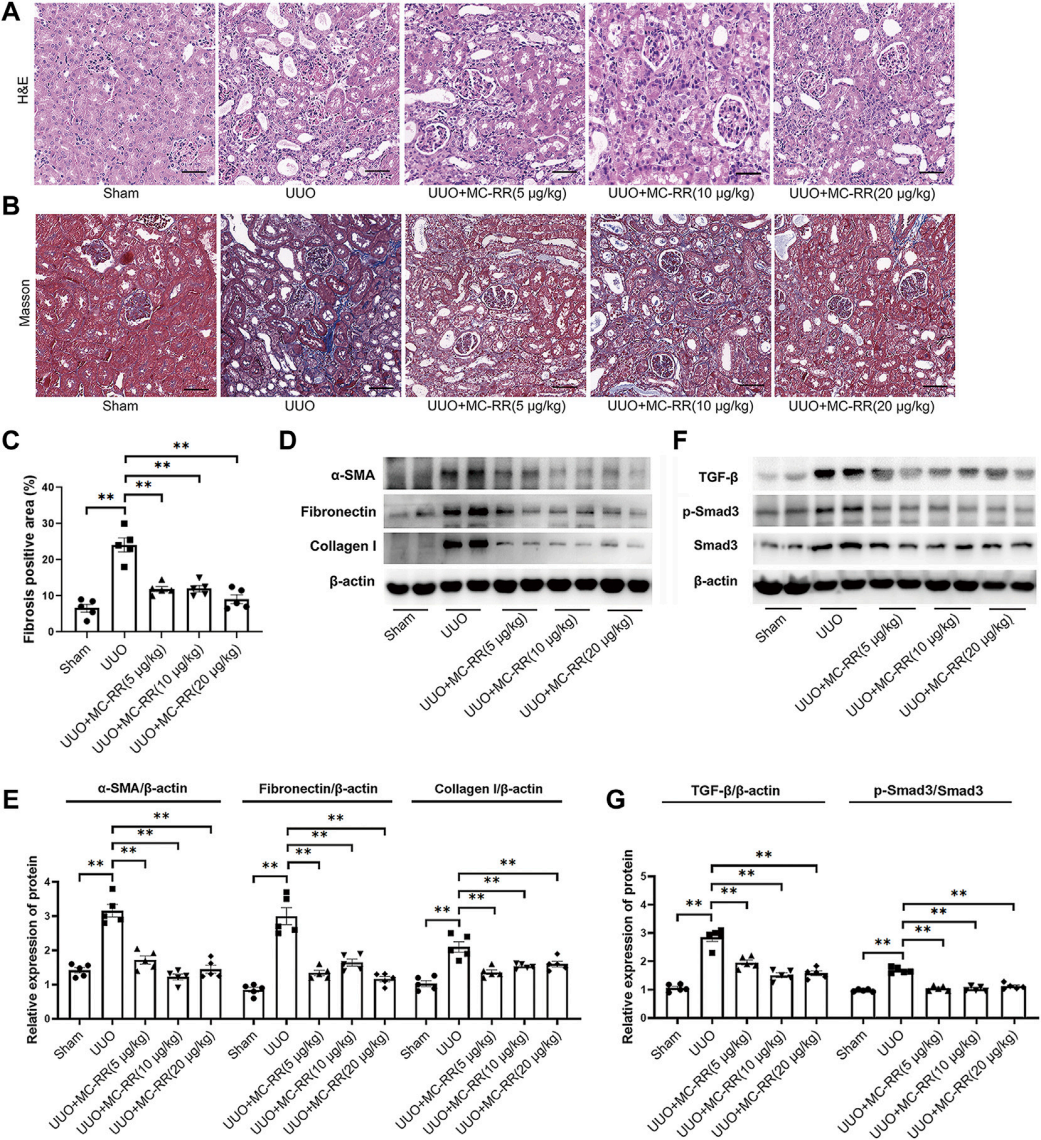
Renoprotection effect of microcystin (MC)-RR in different doses on unilateral ureteral obstruction (UUO) mice. The regimen of MC-RR was used in UUO mice. Twenty-five mice were divided into five groups, Sham, UUO, and UUO mice with treatment of MC-RR in three different doses (5, 10, and 20 μg/kg/day), each group with 5 mice. **(A,B)** Kidney tissue sections were employed for H&E and Masson staining (scale bar: 40 μm). **(C)** Fibrosis positive area in Masson staining were assessed by pathologist blind to this study (*n* = 5). **(D,E)** Protein level of *α*-smooth muscle actin (α-SMA), fibronectin, and collagen I were measured in the renal tissues of UUO mice by western blot. **(F,G)** Protein expression of TGF-β, p-Smad3, and Smad3 in kidney tissue was measured by western blot. *p < 0.05, **p < 0.01 determined by one-way ANOVA with S–N–K *post hoc* analysis.

Based on the fact that no difference in the renoprotection effect was found between MC-LR and MC-RR on UUO mice, the subsequent observations focused on MC-RR for its less toxic and lower tissue residue. We first observed the renoprotection effect of MC-RR administration (20 μg/kg/day) that started at three different times, 14 days, 28 days in advance, and at the day of UUO operation, respectively, and continued until one week after UUO operation. The results indicated that the significant remission of renal fibrosis was observed in the mice with MC-RR treatment starting at 14 and 28 days prior to UUO operation although only a mild remission appeared in the mice starting treatment at the operation day (Supplementary Figure S3). We, then, chose the regimen of MC-RR administration starting at 28 days in advance to evaluate the alleviation of renal fibrosis in three doses (5, 10 or 20 μg/kg/day). The data showed that MC-RR, even at lower concentrations (5 ug/kg/day), substantially alleviated renal injury and interstitial fibrosis deposition in UUO mice (Figures 2A–C). Western blot and immunohistochemistry of kidney tissues indicated that MC-RR significantly suppressed the expression of *α*-SMA, fibronectin, and collagen I in UUO mice (Figures 2D,E, Supplementary Figure S4). Moreover, UUO induced the activation of TGF-β/Smad signaling (Figures 2F,G), and then the upregulated expressions of AKT and STAT6 were also inhibited with MC-RR treatment (Supplementary Figure S5A–C).

### MC-RR Inhibits TGF-β-Induced Epithelial Dedifferentiation and Myofibroblast Differentiation *In Vitro*

**FIGURE 3:**
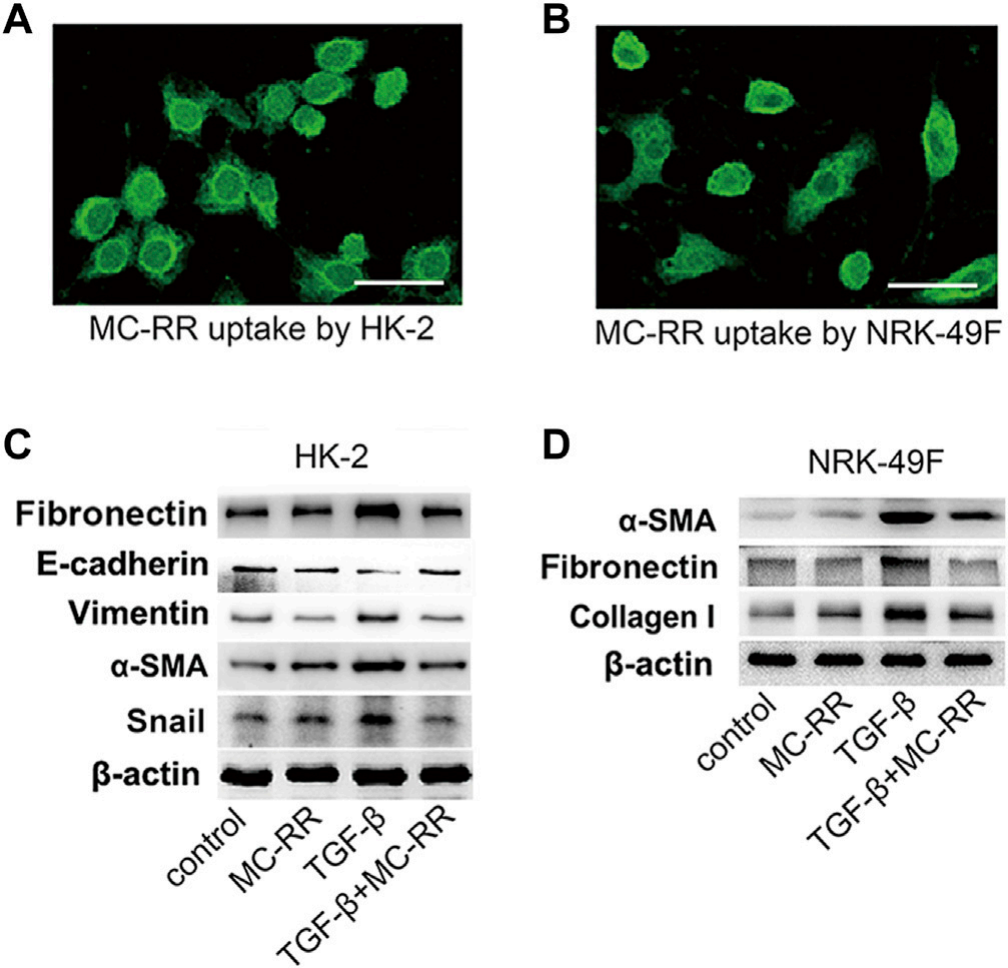
Microcystin (MC)-RR inhibits TGF-β-induced epithelial dedifferentiation and myofibroblast differentiation ***in vitro.* (A,B)** Cultured HK2 (human proximal tubular cell) and NRK-49F (rat kidney fibroblasts) were treated with MC-RR (0.1 μM) for 48 h. The uptake of MC-RR by HK2 and NRK-49F cells was detected by immunofluorescence (scale bar: 40 μm). **(C,D)** HK2 and NRK-49F cells were left alone or cultured with TGF-β1 (5 ng/ml) to induce epithelial dedifferentiation and myofibroblast activation. Some of the cultured cells were also treated with 0.1 μM MC-RR for 48 h. The expression levels of the marker proteins used for assessment of epithelial dedifferentiation and myofibroblast activation in the cultured HK2 or NRK-49F cells were detected by western blot.

We tested whether MC-RR alleviated renal fibrosis via the inhibition of epithelial dedifferentiation and myofibroblast activation *in vitro*. The cell models induced by TGF-β1 showed that the cultured HK-2 and NRK-49F cells had an efficient MC-RR uptake (Figures 3A,B). MC-RR obviously reversed the expression pattern of the partial epithelial to mesenchymal transition cells and activated myofibroblasts, representing the upregulation of E-cadherin, an epithelial marker, and the downregulation of vimentin, snail, fibronectin, *α*-SMA, and collagen I, the marker molecules of myofibroblasts (Figures 3C,D). We also demonstrated that the dose of MC-RR we used here had no inhibition of cell proliferation *in vitro* (Supplementary Figure S6).

### MC-RR Significantly Suppresses the Expression of PKM2 in UUO-Induced Renal Fibrosis

**FIGURE 4:**
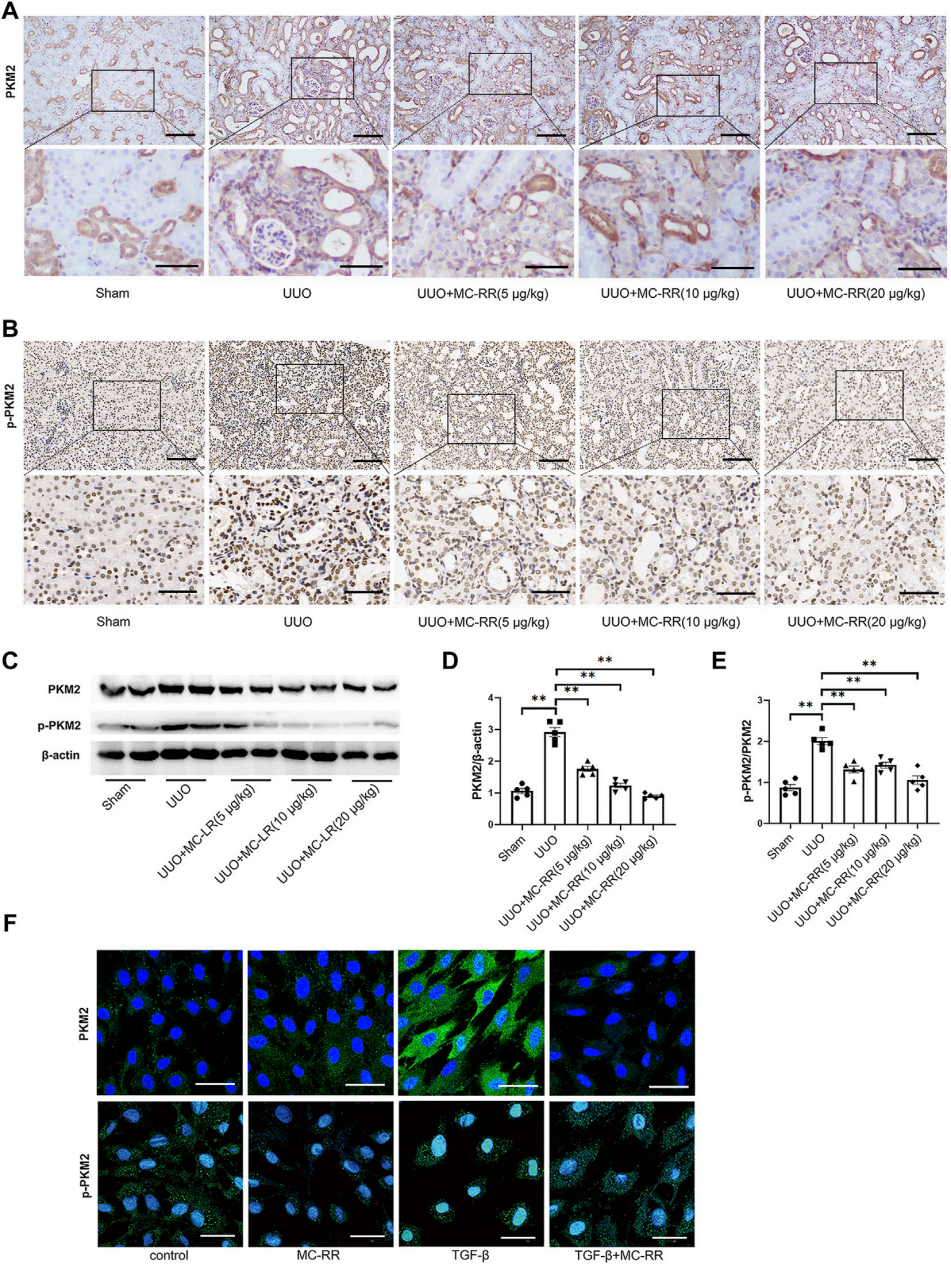
Microcystin (MC)-RR reduces the level of pyruvate kinase M2 (PKM2) and p-PKM2 in the kidney tissues of unilateral ureteral obstruction (UUO) mice and in NRK-49F exposed to TGF-β. UUO mice were treated with MC-RR as described in Figure 2. **(A,B)** Expression levels of PKM2 and p-PKM2 in mice kidney tissue sections were detected by immunohistochemistry (scale bar: 100 and 50 μm). **(C–E)** Total protein was extracted from kidney tissues for determining the expression level of PKM2 and p-PKM2 protein by western blot. *p < 0.05 determined by one-way ANOVA with S–N–K *post hoc* analysis. **(F)** NRK-49F cells were left alone or cultured with TGF-β1 (5 ng/ml) for 48 h. Some of the cultured cells were also treated with 0.1 μM MC-RR. The subcellular localization and protein expression of PKM2 and p-PKM2 were detected by immunofluorescence technique (scale bar: 40 μm).

The changed expression of PKM2 was analyzed in the kidney tissues of UUO mice with MC-RR treatment. The level of p-PKM2 was evaluated synchronously. As expected, the expression of PKM2 was mainly observed in kidney distal tubule cells in the sham mice and the subcellular localization of PKM2 was in the cytoplasm, while p-PKM2 was mainly observed in the nucleus (Figures 4A,B,F). Immunohistochemical staining indicated that both the levels of PKM2 and p-PKM2 were significantly increased in the kidney tissues of the UUO group and observed in renal interstitial cells as well. However, MC-RR treatment remarkably reduced the levels of PKM2 and p-PKM2, which was observed in all three doses (Figures 4A,B). The decreased expression of PKM2 and p-PKM2 mediated by MC-RR was confirmed in western blot data (Figures 4C–E). The cell model *in vitro* was also performed to test the effect of MC-RR on the expression of PKM2 and p-PKM2. The data showed that MC-RR substantially inhibited the PKM2 expression of NRK-49F cells exposed to TGF-β1 and caused a remarkably reduced level of p-PKM2 as well (Figure 4F).

### MC-RR Significantly Inhibits the Expression of HIF-1α and Alters the Levels of Matrix Metalloproteinases (MMPs) in UUO Mice

**FIGURE 5:**
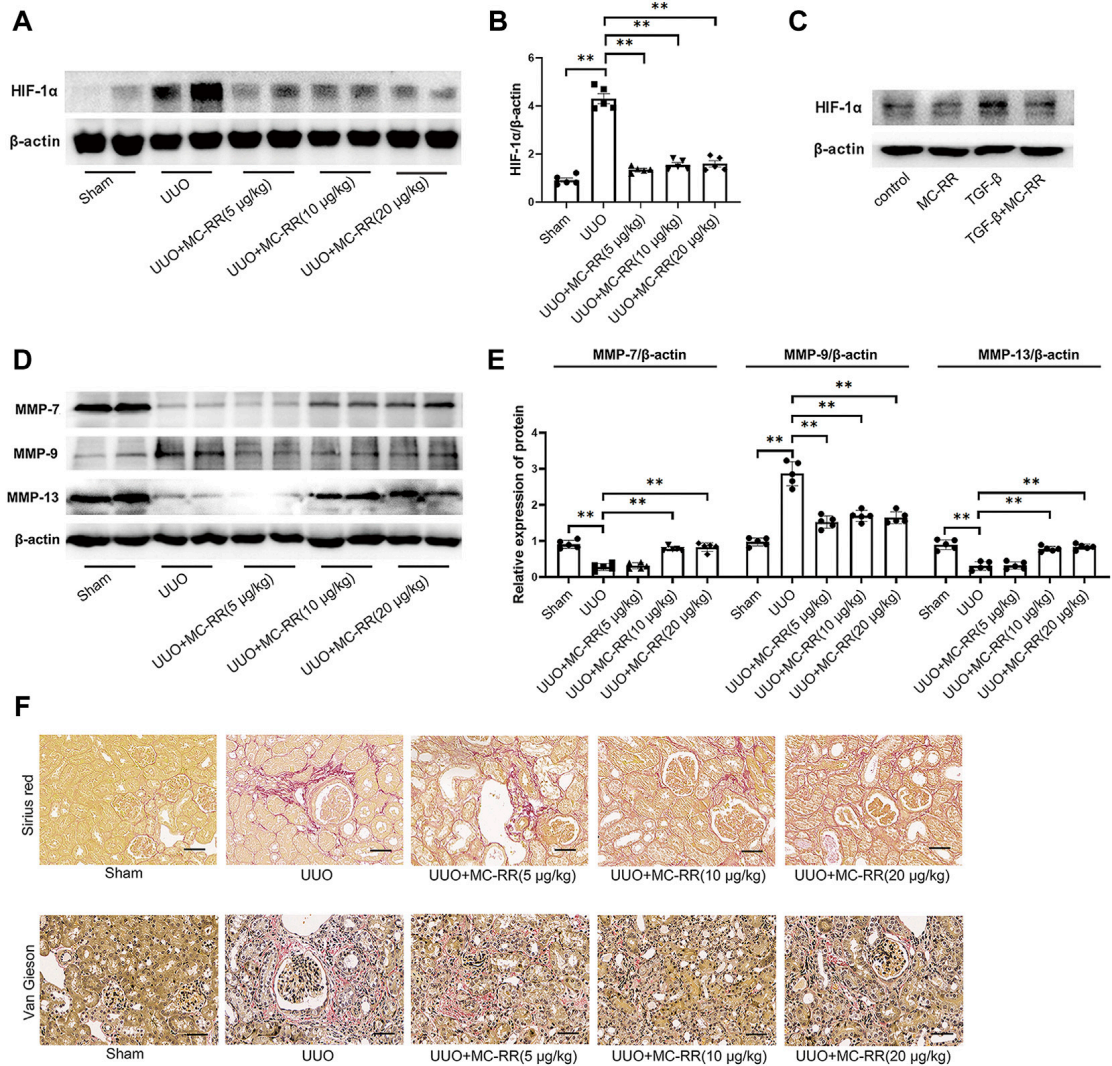
Microcystin (MC)-RR inhibits the expression of hypoxia inducible factor 1 subunit alpha (HIF-1α) and alters the level of matrix metalloproteinases (MMPs). Unilateral ureteral obstruction (UUO) mice and the cultured NRK-49F cells were treated as described in Figure 2 and Figure 4, respectively. **(A,B)** Expression of HIF-1α protein in the kidney tissues was detected by western blot. **(C)** Level of HIF-1α in NRK-49F cells was determined by western blot. **(D,E)** Expression levels of MMP-7, MMP-9, and MMP-13 in the kidney tissues of UUO mice were analyzed by western blot. **(F)** Deposition of collagen and elastic fibers in the kidney tissues of UUO mice with MC-RR treatment was evaluated by Sirius Red staining and Van Gieson staining (scale bar: 40 μm). *p < 0.05, **p < 0.01 determined by one-way ANOVA with S–N–K *post hoc* analysis, *n* = 5.

We analyzed the expression of HIF-1α in the model mice with MC-RR treatment and found that MC-RR significantly suppressed the increased expression of HIF-1α induced by UUO, even in the treatment with a lower dose (5 μg/kg/day) (Figures 5A,B). The experiment *in vitro* supported the suppression effect of MC-RR on the expression of HIF-1α in TGF-β-exposed NRK-49F cells (Figure 5C). Numerous pieces of evidence indicate that the balanced expression of MMPs maintains the stability of the interstitial structure. Therefore, we evaluated the expression of MMP-7 (matrilysin), MMP-9 (gelatinase B), and MMP-13 (collagenase 3) in the kidney tissues of UUO mice with MC-RR treatment. The data showed a significantly increased expression of MMP-9 and the decreased expressions of MMP-7 and MMP-13 were associated with UUO-induced renal fibrosis. Interestingly, MC-RR treatment obviously reversed these altered expressions of MMPs, presenting a reduced MMP-9 and an elevated MMP-7 and MMP-13 when compared with the UUO group (Figures 5D,E). Corresponding to that, Sirius Red and Elastica-van-Gieson (EVG) staining showed a remarkable accumulation of collagen and elastic fibers in the UUO group, which was lightened with MC-RR treatment (Figure 5F). We also assessed the expression of M2 macrophage markers, including CD206, Arg1 and Ym1, and inducible nitric oxide synthase (iNOS), a marker of M1 macrophages in the kidney tissue of UUO mice. The results showed that MC-RR inhibited the UUO-induced upregulation of M2 macrophage markers, but had no obvious impact on iNOS (Supplementary Figure S7).

### MC-RR Inhibits the Expression of PKM2 by an Interaction Between the Two Molecules

**FIGURE 6:**
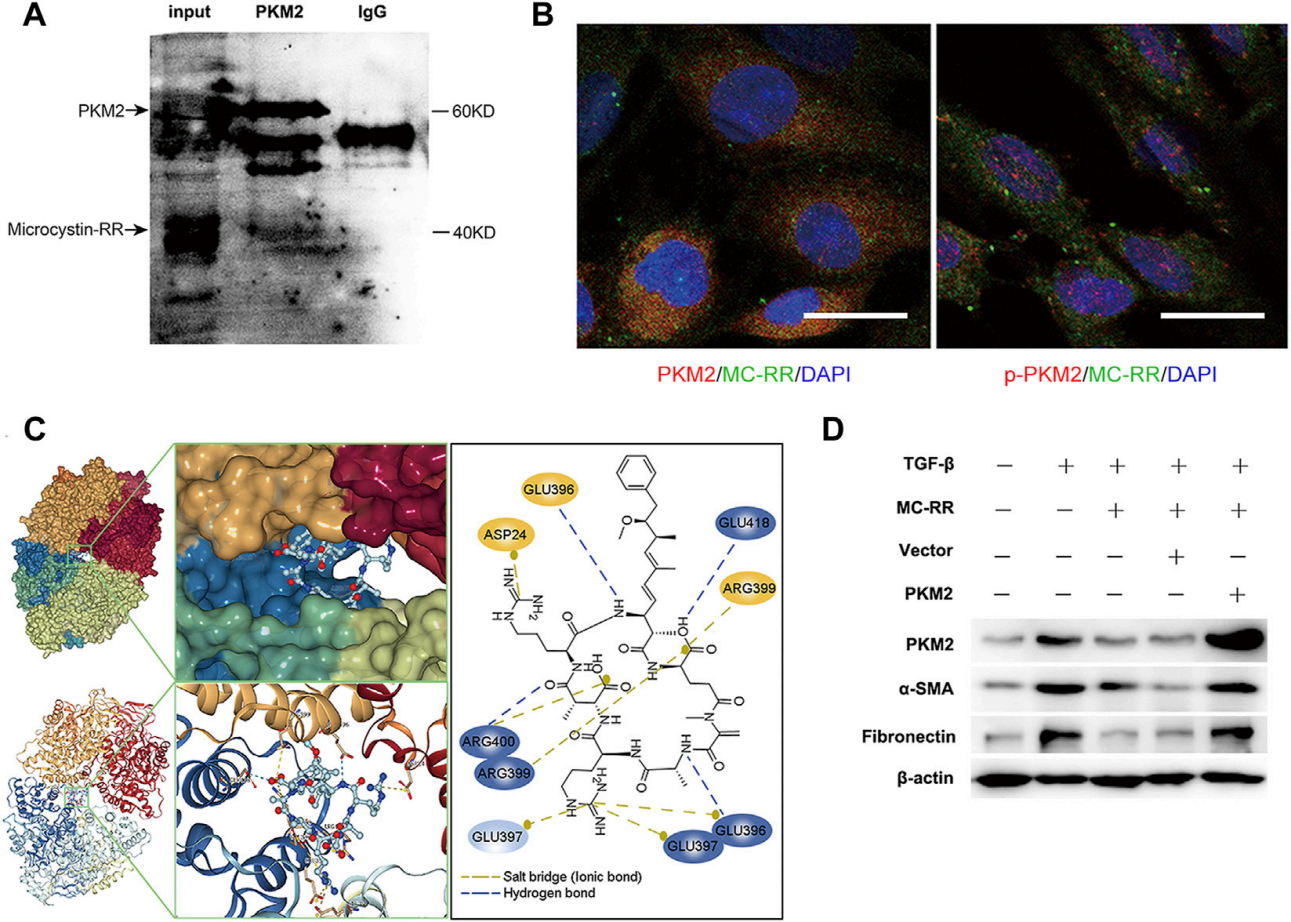
Microcystin (MC)-RR reduces the level of pyruvate kinase M2 (PKM2) via interaction with PKM2. NRK-49F cells were treated as explained in Figure 4. **(A)** Total protein extracted from the cultured NRK-49F cells pretreated with TGF-β1 and MC-RR was immunoprecipitated with the PKM2 antibody. A protein band was subsequently identified as MC-RR by western blot. **(B)** Examination on colocalization of MC-RR (green) with PKM2 (red) or with p-PKM2 (red) was performed by immunofluorescence staining. DAPI was used for nuclear staining (blue) (scale bar: 20 μm). **(C)** Interaction between PKM2 and MC-RR was predicted with molecular docking conducted using the Yinfo Cloud Platform. The hydrogen bonds (showed in broken blue line) and ionic bonds (in broken yellow line) were formed between the amino acid residues of PKM2 and MC-RR. **(D)** PKM2 rescue assay was performed by transfecting NRK-49F cells with the recombinant PKM2 expression plasmid (pcDNA3.1-PKM2). Control cells without transfection, vector (pcDNA3.1), and the pcDNA3.1-PKM2 transfected cells were treated as indicated for 48 h. The expressions of PKM2, *α*-smooth muscle actin (α-SMA), and fibronectin were detected by western blot.

With the cultured NRK-49F cells pretreated with TGF-β1 and MC-RR, we conducted the assay of immunoprecipitation to explore the mechanisms of MC-RR–mediated inhibition of PKM2. Interestingly, MC-RR showed an interaction with PKM2 by binding to this target protein (Figure 6A). Immunofluorescence technique also showed a strong overlap of MC-RR (green) with PKM2 (red) in the subcellular localization of the cytoplasm, rather than with p-PKM2 (red) in the nucleus (Figure 6B). To further characterize the interaction between MC-RR and PKM2, microscale thermophoresis was performed to test the binding affinity of MC-RR with monomer PKM2. But no interaction was observed between the two molecules (Supplementary Figure S8). Given that PKM2 with allosteric regulation exists as either a dimer with non-glycolytic function or tetramer with the glycolytic function, we next performed the molecular docking study to simulate the binding affinity of MC-RR with either the dimer or tetramer PKM2. The dimer and tetramer PKM2 were built in 3D structures, respectively, and each monomer in them was marked in different colors (red, yellow, green, and blue). Molecular docking studies revealed a stronger binding affinity between MC-RR and tetramer PKM2 (Figure 6C, left). MC-RR is bound to tetramer PKM2 by hydrogen bonds (broken blue line) and ionic bonds (broken yellow line) between the amino acid residues of the two molecules (Figure 6C, right). Three monomers (yellow, green, and blue) of tetramer PKM2 were involved in the formation of hydrogen and/or ionic bonds) with MC-RR (Figure 6C). Moreover, the overexpression of PKM2 with the recombinant plasmid (pcDNA3.1-PKM2) restored the expression of *α*-SMA and fibronectin, which was inhibited by MC-RR treatment *in vitro* (Figure 6D). On the other hand, we used TEPP-46, a synthetic small-molecule activator that enhance the formation of the PKM2 tetramer, to treat the cultured NRK-49F cells and observed a reduced expression of PKM2 induced by TGF-β1 exposure (Supplementary Figure S9).

### MC-RR Treatment Does Not Cause Extra Damage of the Liver and Kidney Function in UUO Mice

To investigate whether MC-RR treatment (20 μg/kg/day) in the current study caused extra damage of the liver and kidney function in UUO mice, we performed an assay of blood samples. The data exhibited no observable adverse effect on serum biochemistry with the highest dose of MC-RR treatment in this study (Supplementary Table S1).

## Discussion

We previously unveiled the antifibrotic function of MCs, involving the more toxic MC-LR and less toxic MC-RR, which reduced TGF-β1/Smad signaling and blocked epithelial dedifferentiation and myofibroblast activation by suppressing the polarization of CD206 + macrophages (Wang et al., 2020; Wang et al., 2021). MC-LR and MC-RR are hydrophilic and generally incapable of crossing cell membranes via passive diffusion. In fact, MCs enter the cells by the active transport through specific transporters, an organic anion–transporting polypeptides (rodent Oatp/human OATP) family (Fischer et al., 2005; Fischer et al., 2010). A wide variation of Oatp/OATP expression in type and level has been well demonstrated among tissue cells (Kalliokoski and Niemi, 2019). Therefore, the antifibrotic characteristics of MCs on renal fibrosis were investigated in the current study.

The rodent model of UUO is widely used to elucidate mechanisms responsible for progressive renal fibrosis as well as a new therapeutic strategy for this disease (Yang et al., 2010). The complete UUO initiates a rapid sequence of events in the obstructed kidney, leading to renal blood flow and metabolic changes within 24 h, tubular injury and interstitial macrophage infiltration in 2–3 days, and interstitial fibrosis in 7 days (Chevalier et al., 2009). Based on the course taken in the management of CKD patients, we chose the regimen that started the intervention of MCs four weeks before UUO implement in relatively low three doses by oral administration to investigate the renoprotection mechanism.

The toxicity of MC-RR has been well known to be less than 1/10 of that of MC-LR, but MC-RR represented a similar renoprotection function as MC-LR in UUO mice. Compared with MC-LR, MC-RR showed a lower accumulation in renal tissues after treatment, which is obviously conducive to transformation in therapy of chronic kidney diseases. The current study focused on the renoprotection of MC-RR and demonstrated that MC-RR treatment, even in a lower dose, substantially alleviated the main characteristic phenotypes of renal fibrosis and reduced the expression levels of the marker molecules of dedifferentiated epithelial cells and activated fibroblasts, indicating the inhibition of myofibroblast differentiation. We previously reported that the therapeutic effect of MC-LR on the amelioration of pulmonary fibrosis was achieved through modulating CD206^+^ M2-like macrophages (Wang et al., 2020). In contrast to lung epitheliums and fibroblasts, the cultured HK2 and NRK-49F represented an efficient MC-RR uptake. Moreover, MC-RR exerts a direct effect on the renal epithelial cells and fibroblasts to block epithelial dedifferentiation and myofibroblast activation with TGF-β1 exposure, suggesting a different mechanism for the therapeutic effect of MC-RR between pulmonary and renal fibrosis. The Oatp/OATP family consists of 11 members that have differential tissue distribution and varying affinities for MCs. As a result, the systemic distribution and toxicity of MCs are governed by the type and expression level of Oatp/OATP on the cell membranes of a given organ (Díez-Quijada et al., 2019). Therefore, we further identified the unknown mechanism of MC-RR alleviating renal fibrosis with the UUO model.

PKM2, the embryonic isoform of pyruvate kinase, has been known as a crucial regulator of aerobic glycolysis for the Warburg effect. PKM2 is also expressed in cancer and some normal tissue cells, for instance, kidney distal tubule cells (Dayton et al., 2016). Interestingly, a significantly increased expression of PKM2 was expectedly observed both in tubules and interstitial cells in UUO animals, but the intervention of MC-RR substantially reversed the high level of PKM2 associated with renal fibrosis. This result strongly suggests that MC-RR can effectively suppress the expression of PKM2. Increasing evidence shows that PKM2 is allosterically regulated to form either a tetramer with glycolytic functions or a dimer in phosphorylation with nonglycolytic functions (Donovan et al., 2016; Schormann et al., 2019). The p-PKM2 in the cytoplasm can translocate into the nucleus and combine with HIF-1α as a transcriptional cofactor in there, as a result, endows the cells with a survival and proliferative advantage. The pathway of PKM2-HIF-1α provides a feedforward mechanism that amplifies HIF-1α dependent metabolic reprogramming and maintains the expression of PKM2 at a high level (Higgins et al., 2007; Tamada et al., 2012; Palsson-McDermott et al., 2015). In line with the decreased PKM2, the level of HIF-1α was considerably reduced with MC-RR treatment while it was obviously upregulated in renal fibrogenesis. Consistently, p-PKM2 showed a significant downregulation and was dose-dependent with MC-RR intervention. This result clearly supports that the suppressed PKM2 plays a critical role in the renoprotection of MC-RR in UUO animals. Furthermore, we observed an interaction between MC-RR and PKM2. The subcellular localization showed a strong overlap of MC-RR with PKM2 in the cytoplasm but not with p-PKM2 in the nucleus. Consistently, the molecular docking calculation indicates that MC-RR binds strongly to tetrameric PKM2. It is reasonable to conceive that the interaction between MC-RR and tetrameric PKM2 can initiate a switch of PKM2 from dimer to tetramer formation, which prevents the dimer from entering the nucleus, blocks the PKM2-HIF-1α pathway, and ultimately results in the reduction of PKM2 expression. This hypothesis is supported by the observation on TEPP-46-associated suppression of PKM2. TEPP-46 is a small-molecule activator that can bind to a pocket at the PKM2 subunit interface and induces PKM2 into a stable tetramer (Anastasiou et al., 2012).

ECM is a three-dimensional macromolecular network composed of collagens, elastin, fibronectin, and several other glycoproteins, and is a highly dynamic structure with a continuous remodeling under either normal or pathological circumstances (Theocharis et al., 2016; Amar et al., 2017; Bülow and Boor, 2019). MMPs are involved in the mediation of matrix degrading and remodeling (Craig et al., 2015; Freitas-Rodríguez et al., 2017). Evidence supports that the altered expression of MMPs can be caused by the inhibition of HIF-1α and aerobic glycolysis (Sakamoto et al., 2011; Conde et al., 2017). Special histology staining for the visualization of ECM showed an observable accumulation of collagen and elastic fibers in the renal tissue of UUO animals. MC-RR treatment significantly reduced both collagen and elastin deposition. We also revealed that the levels of MMP-7 and MMP-13 were partially restored while MMP-9 was inhibited with MC-RR treatment in UUO animals. MMP-13 has been considered as an antifibrosis molecule in that the expression of MMP-13 in model animals contributes to the resolution of fibrosis (Cabrera et al., 2019; Ren et al., 2019), whereas the collagen fragments produced by MMP-9 are chemotactic for neutrophils and MMP-9 deficiency decreased kidney fibrosis in murine UUO (Wang et al., 2010; Wang et al., 2019). Matrisian LM et al. showed that MMP-7 is the predominant MMP with multiple functions in both the intact and injured lung, one of which is to facilitate re-epithelialization (Dunsmore et al., 1998). The restored expression of MMP-7 following MC-RR treatment could be beneficial for the reduction of lesion in UUO animals (Liu et al., 2020).

In the previous intervention study of MC-LR on bleomycin-induced pulmonary fibrosis, we first reported that MC-LR ameliorated pulmonary fibrosis through a blockage of endoplasmic reticulum stress (ERS) signaling and modulation of CD206^+^ M2-like macrophage polarization. The current study observed a decreased expression of the marker molecules of M2 macrophages, including CD206, Arg1, and YM1 in the renal tissues of UUO animals with MC-RR treatment, indicating that attenuation of macrophage M2 polarization plays a role in MC-RR-associated renoprotection as well. Further investigation is needed to clarify the cross-talk between ERS-associated attenuation of M2 polarization and inhibition of PKM2 expression mediated by MC-RR (Li et al., 2015). Our data provide a novel effect of MC-RR on the renoprotection of UUO-induced renal fibrosis by inhibiting PKM2-HIF-1α signaling, which highlights its potential application in chronic kidney disease treatment.

## Data Availability

The original contributions presented in the study are included in the article/Supplementary Material; further inquiries can be directed to the corresponding author.
