# Supplementary material for: Renoprotection of Microcystin-RR in Unilateral Ureteral Obstruction-Induced Renal Fibrosis: Targeting the PKM2-HIF-1α Pathway
Source: Front Pharmacol. 2022 Jun 9;13:830312. doi: 10.3389/fphar.2022.830312 (PMC9218570; doi:10.3389/fphar.2022.830312)
Supplement: Supplementary file 1 [file DataSheet1.docx]

**Supplementary Materials**

**Contents**

**Supplementary Figure 1.** Body weights and kidney weight coefficient of the model mice.

**Supplementary Figure 2.** Microcystin (MC) inhibited the expression levels of α smooth muscle actin (α-SMA), fibronectin and collagen I in unilateral ureteral obstruction (UUO)-induced kidney tissues.

**Supplementary Figure 3.** Renoprotection effect of microcystin-RR treatment starting at different times in advance on unilateral ureteral obstruction (UUO) mice.

**Supplementary Figure 4.** Microcystin (MC)-RR decreased the expression of the fibrosis-related proteins.

**Supplementary Figure 5.** Microcystin (MC)-RR blocks the activation of AKT and signal transducer and activator of transcription 6 (STAT6) signal.

**Supplementary Figure 6.** The regimen of microcystin (MC)-RR used for renoprotection dose not inhibit cell proliferation.

**Supplementary Figure 7.** Microcystin (MC)-RR alters unilateral ureteral obstruction (UUO)-induced CD206^+^ M2 subtype of macrophages polarization.

**Supplementary Figure 8.** Test on the binding affinity between pyruvate kinase M2 (PKM2) and microcystin (MC)-RR with microscale thermophoresis (MST).

**Supplementary Figure 9.** TEPP-46 reduces the level of pyruvate kinase M2 (PKM2) in NRK-49F cells exposed to TGF-β.

**Supplementary Table 1.** Serum biochemistry parameters of model mice.

**Supplementary figure and figure legends**


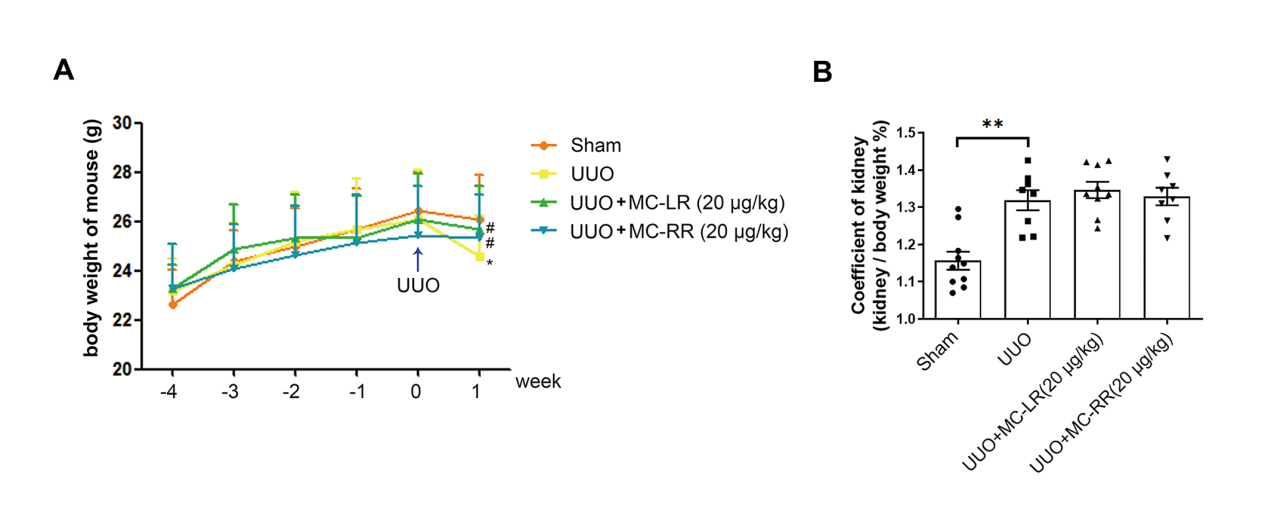


**Supplementary Figure 1 Body weights and kidney weight** **coefficient of the model mice**. Mice were treated as description in Figure 1. (**A**) The experimental mice were weighed every week. Data are presented as mean ± SEM. Comparison between the sham and unilateral ureteral obstruction (UUO) groups, **P* < 0.05. Comparison between UUO mice and UUO + microcystin (MC)-LR/UUO + MC-RR groups, #*P* < 0.05. (**B**) The experimental mice were euthanized one week after UUO. The kidney weight coefficients were measured to make a comparison among the mice with different treatment. Data were presented as mean ± SEM. Comparison between the sham and UUO groups, ***P* < 0.01.

**
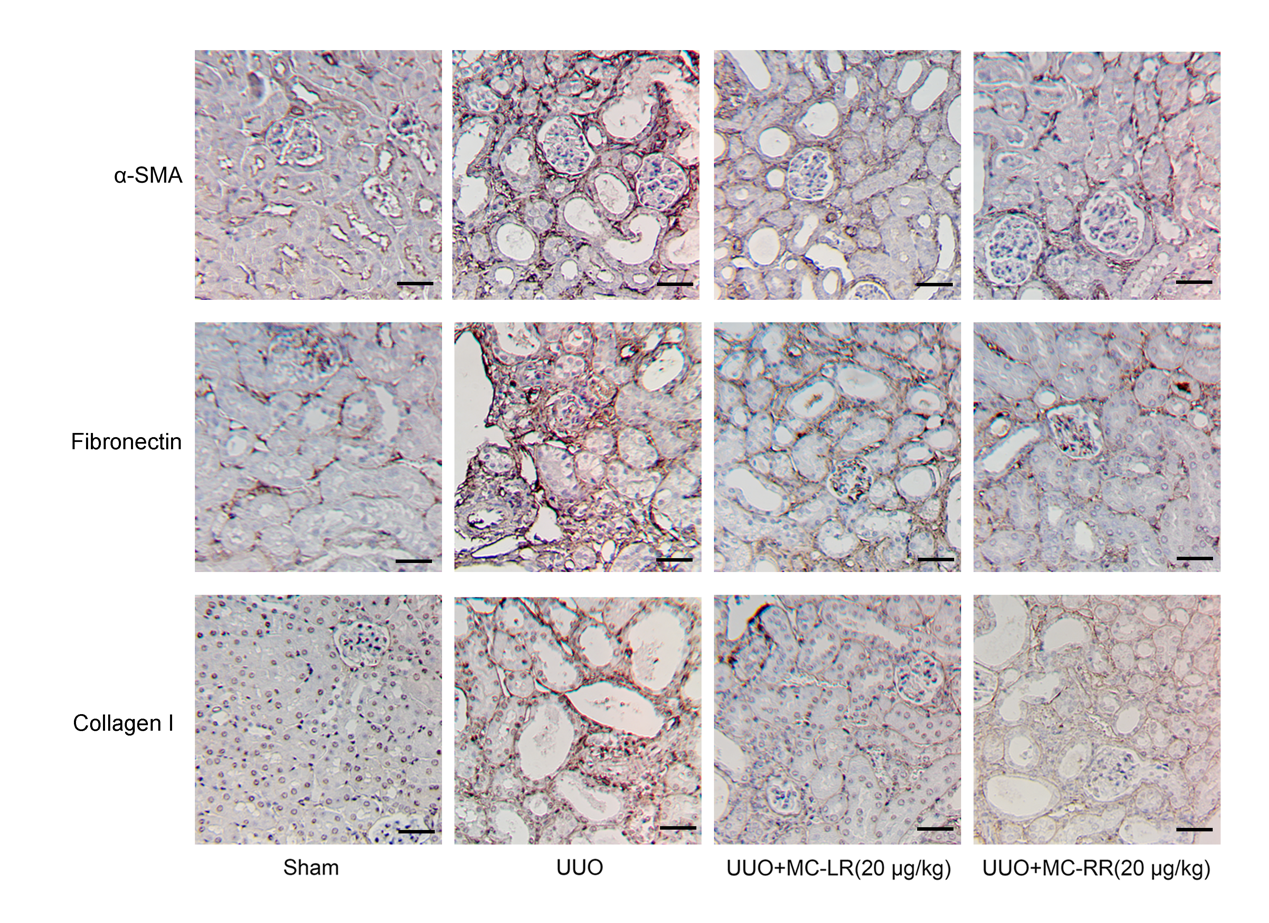
**

**Supplementary Figure 2 Microcystin (MC) inhibited the expression levels of α smooth muscle actin (α-SMA), fibronectin and collagen I in the kidney tissues of unilateral ureteral obstruction (UUO) mice.** Mice were treated with MC as described in Figure 1. The expression level of α-SMA, fibronectin and collagen I in UUO-induced kidney tissues were detected by immunohistochemistry (Scale bar: 40 μm; n=6).


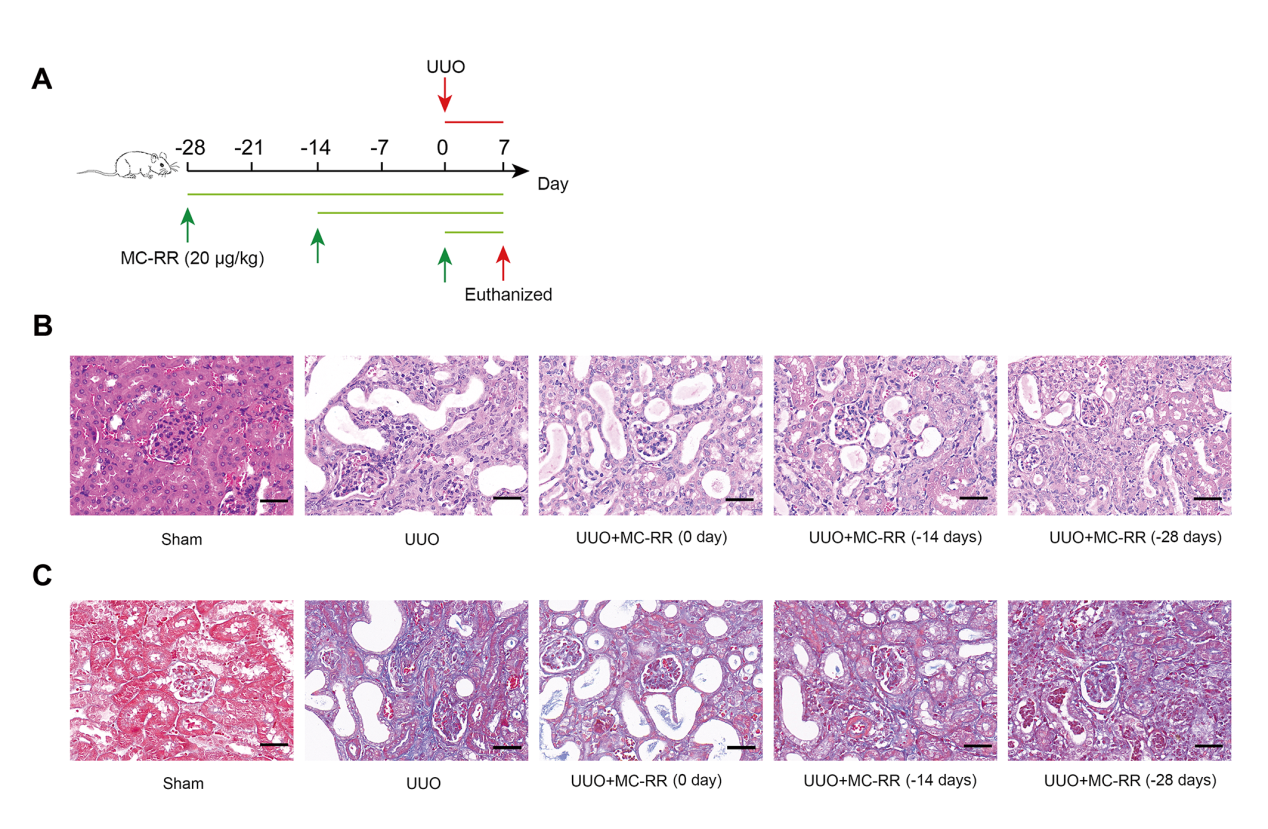


**Supplementary Figure 3 Renoprotection effect of microcystin-RR treatment starting at different times in advance of unilateral ureteral obstruction (UUO) on mice**. Mice were treatment with MC-RR (20 μg/kg/day) by intragastrical administration for 0 day, 14 days and 28 days in advance, and then unilateral ureteral ligation was performed to construct a mouse model of obstructive renal fibrosis. The operated mice were administrated with MC-RR for another week, and then were euthanized for further analysis. (**A**) The schematic diagram of the experimental design. (**B** and **C**) The kidney tissue sections were employed for H&E and Masson staining (Scale bars: 50 μm, n=10).

**
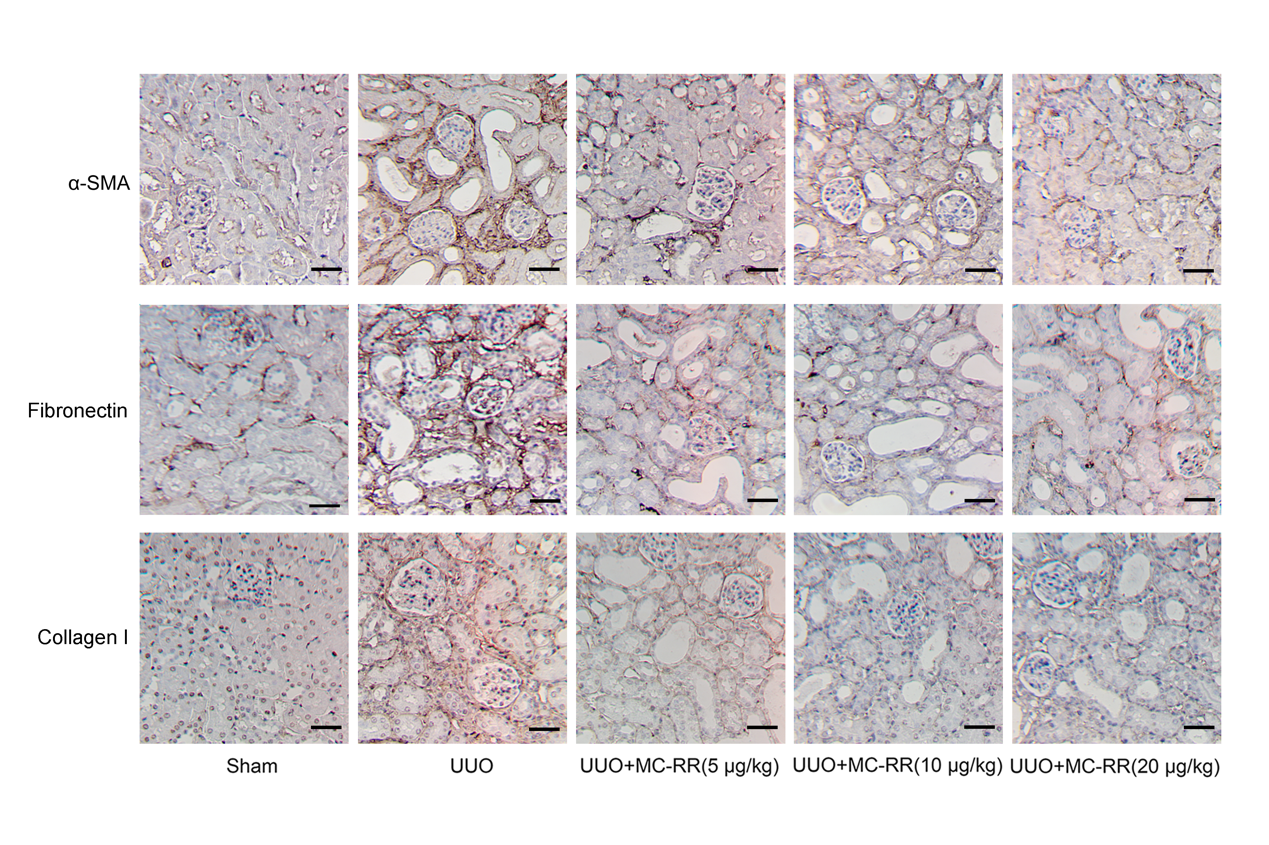
**

**Supplementary Figure 4** **Microcystin (MC)-RR decreased the expression of the fibrosis-related proteins in model mice.** Unilateral ureteral obstruction (UUO) mice were treated with MC-RR as described in Figure 2. The expression level of α smooth muscle actin (α-SMA), fibronectin and collagen I in the kidney tissues of model mice were detected by immunohistochemistry (Scale bar: 40 μm; n=5).


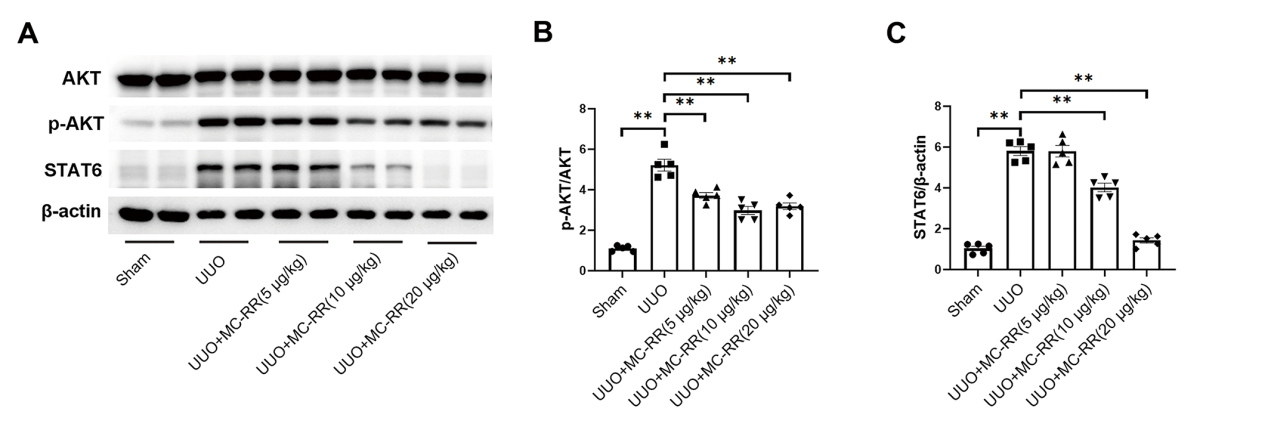


**Supplementary Figure 5 Microcystin (MC)-RR blocks the activation of AKT and signal transducer and activator of transcription 6 (STAT6) signal.** The unilateral ureteral obstruction (UUO) mice were treated as description in Figure 2. (**A, B** and **C**) The expressions of p-AKT, AKT and STAT6 in the kidney tissues of model mice were measured by western blot. * *P*< 0.05, ***P* < 0.01 determined by one-way ANOVA with S-N-K post-hoc analysis (*n*=5).


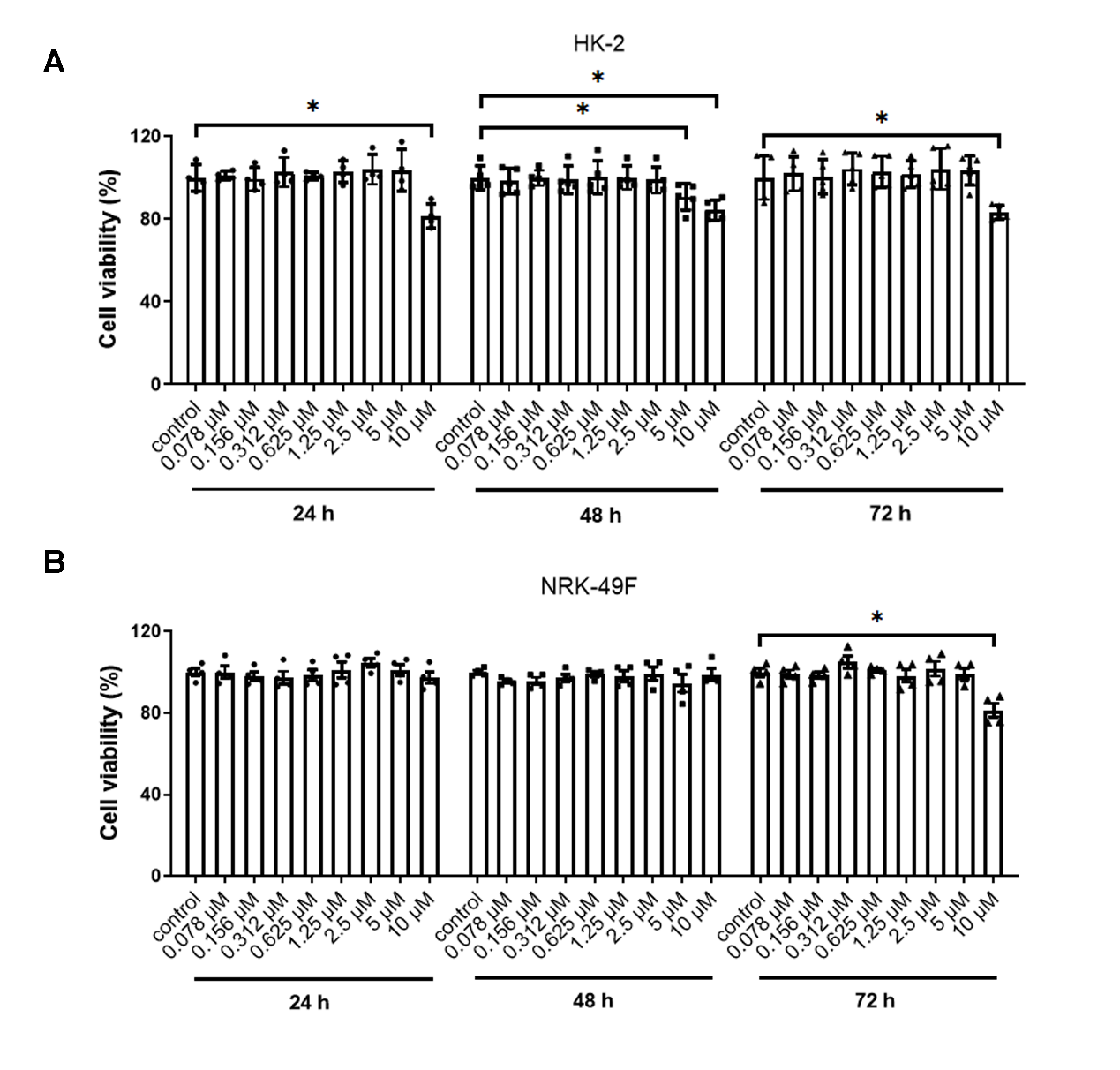


**Supplementary Figure 6 The regimen of microcystin (MC)-RR used for renoprotection dose not inhibit cell proliferation.** (**A** and **B**) Renal cells (HK-2 and NRK-49F) were treated with the indicated concentrations of MC-RR for 24, 48 and 72 h, respectively. Cell viability was measured by MTT assay. Data are normalized to MC-RR untreated control group. * *P*< 0.05 determined by one-way ANOVA with S-N-K post-hoc analysis (*n*=4).


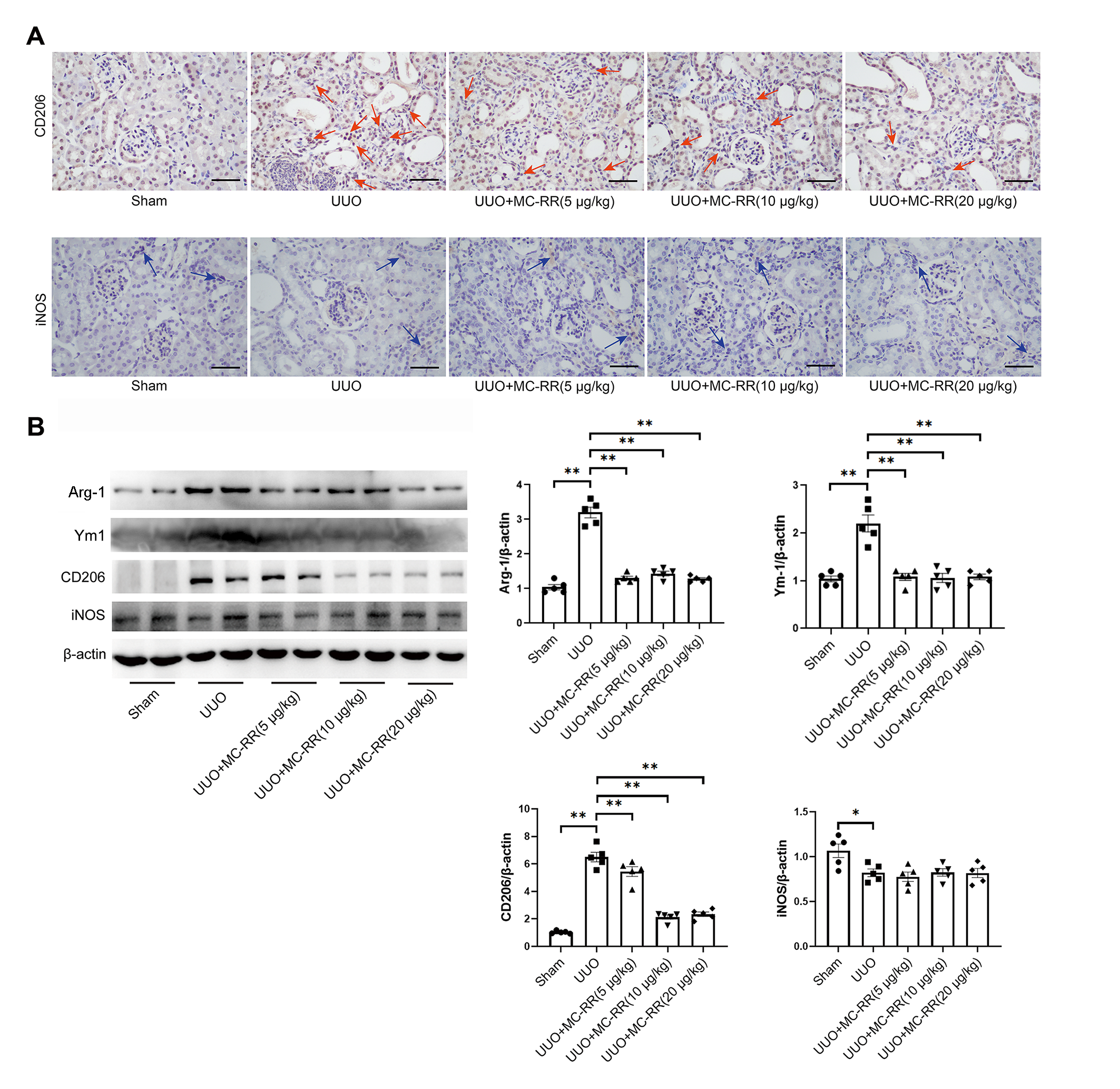


**Supplementary Figure 7 Microcystin (MC)-RR alters unilateral ureteral obstruction (UUO)-induced CD206^+^ M2 subtype of macrophages polarization.** The UUO mice were treated as description in Figure 2. (**A**) The kidney tissue sections of model mice were examined for the expression of CD206 (red arrows), a marker of M2a macrophages, and inducible nitric oxide synthase (iNOS) (blue arrows), a marker as M1 macrophages by immunohistochemistry (Scale bars: 50 μm). (**B**) Total protein was extracted from mice kidney tissues and evaluated for the expression of arginase-1 (Arg1), chitinase-like 3 (Ym-1), CD206 and iNOS using western blot. **P* < 0.05, ***P* < 0.01 was determined by one-way ANOVA with S-N-K post-hoc analysis.


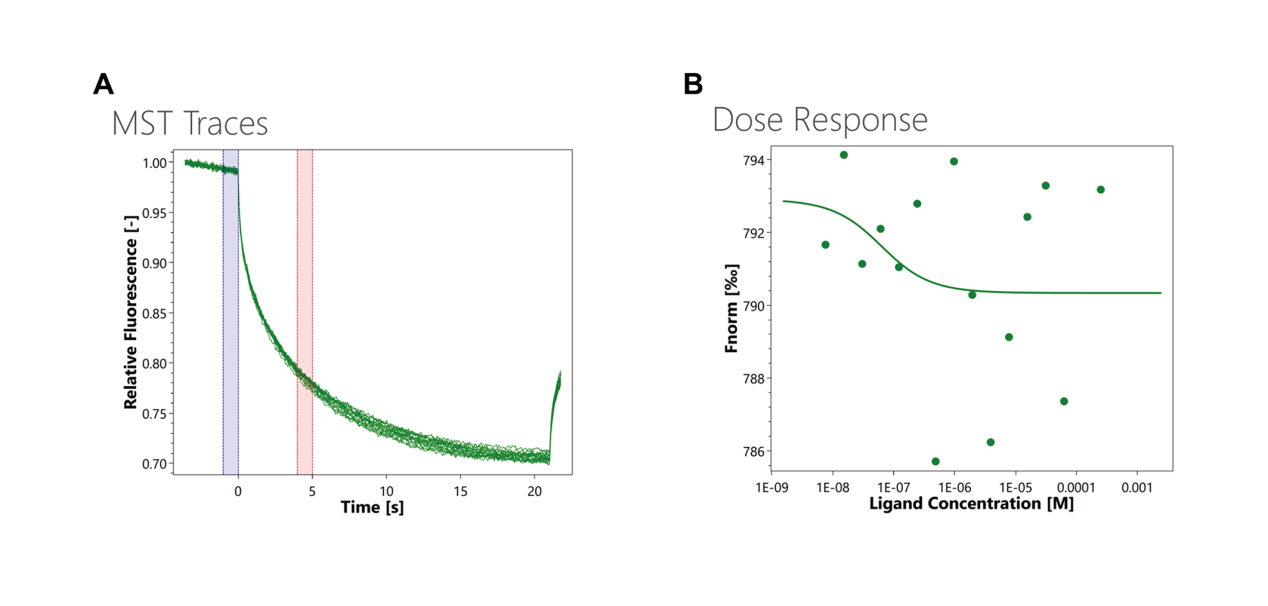


**Supplementary Figure 8 Test on the binding affinity between pyruvate kinase M2 (PKM2) and microcystin (MC)-RR with microscale thermophoresis (MST).** (**A** and **B**) The binding affinity between MC-RR and PKM2 monomer was detected using MST assay.


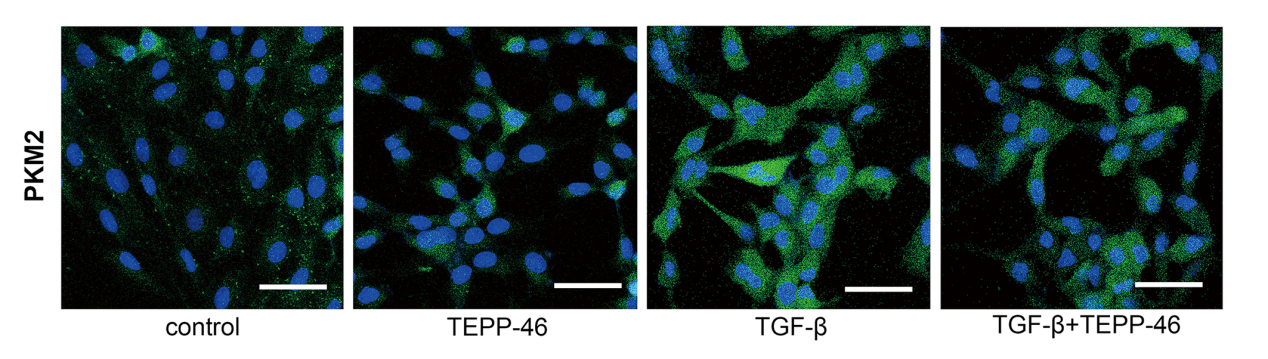


**Supplementary Figure 9 TEPP-46 reduces the level of pyruvate kinase M2 (PKM2) in NRK-49F cells exposed to TGF-β.** NRK-49F cells were left alone or cultured with TGF-β1 (5 ng/ml) to induce the expression of PKM2. Some of the cultured cells were also treated with TEPP-46 (50 μM) for 48 h. The expression levels of PKM2 in the cultured NRK-49F cells were detected by immunofluorescence technique (scale bar: 40 μm).

| **Supplementary Table 1. Serum biochemistry parameters of model mice.** | | | | | | |
| --- | --- | --- | --- | --- | --- | --- |
| Treatment | ALT (UI/L) | AST (UI/L) | TP (g/L) | TB (μmol/L) | BUN (mmol/L) | CRE (μmol/L) |
| Sham | 21.70 ± 1.10 | 88.77 ± 6.72 | 46.84 ± 0.69 | 6.33 ± 0.15 | 9.13 ± 0.26 | 30.05 ± 0.63 |
| UUO | 18.34 ± 0.61 | 83.03 ± 2.00 | 45.94 ± 1.04 | 5.25 ± 0.22 | 9.03 ± 0.53 | 29.94 ± 1.82 |
| UUO + MC-RR | 19.48 ± 1.23 | 84.72 ± 4.17 | 45.76 ± 0.80 | 6.09 ± 0.18 | 9.11 ± 0.31 | 28.87 ± 1.44 |
| Mice were treated as explained in Figure 1. Serum biochemical parameters were measured in the Sham, UUO and UUO + MC-RR (20 μg/kg/day) groups. Data are presented as mean ± SEM and analyzed by one-way ANOVA with S-N-K post-hoc analysis (*n*=10). ALT, alanine aminotransferase; AST, aspartate aminotransferase; TP, total protein; TB, total bilirubin; BUN, blood urea nitrogen; CRE, creatinine. | | | | | | |
